# On the Stochastic Motion Induced by Magnetic Fields in Random Environments

**DOI:** 10.3390/e27040330

**Published:** 2025-03-21

**Authors:** Yun Jeong Kang, Jae Won Jung, Sung Kyu Seo, Kyungsik Kim

**Affiliations:** 1 School of Liberal Studies, Wonkwang University, Iksan 54538, Republic of Korea; yjkang66@wku.ac.kr; 2 DigiQuay Company Ltd., Seoul 06552, Republic of Korea; hotliver@gmail.com (J.W.J.); ggaru79@gmail.com (S.K.S.); 3 Department of Physics, Pukyong National University, Busan 48513, Republic of Korea

**Keywords:** Fokker–Planck equation, Navier–Stokes equation, passive particle, joint probability density, super diffusion, magnetic field

## Abstract

Here, we study the Navier–Stokes equation for the motion of a passive particle based on the Fokker–Planck equation in an incompressible conducting fluid induced by a magnetic field subject to an exponentially correlated Gaussian force in three-time domains. For the hydro-magnetic case of velocity and the time-dependent magnetic field, the mean squared velocity for the joint probability density of velocity and the magnetic field has a super-diffusive form that scales as $∼t^{3}$ in t>>τ, while the mean squared displacement for the joint probability density of velocity and the magnetic field reduces to time $∼t^{4}$ in t<<τ. The motion of a passive particle for τ=0 and t>>τ behaves as a normal diffusion with the mean squared magnetic field being $<h^{2}(t)>∼t$. In a short-time domain t<<τ, the moment in the magnetic field of the incompressible conducting fluid undergoes super-diffusion with $\mu_{2,0,2}^{h}∼t^{6}$, in agreement with our research outcome. Particularly, the combined entropy H(v,h,t) (H(h,v,t)) for an active particle with the perturbative force has a minimum value of $∼\ln t^{2}$ ($∼\ln t^{2}$) in t>>τ (τ=0), while the largest displacement entropy value is proportional to $\ln t^{4}$ in t<<τ and τ=0.

## 1. Introduction

Past studies have utilized the Navier–Stokes equation to model fluid motion, incorporating viscosity and pressure terms to capture the intricate dynamics. The Navier–Stokes equation considers viscous flow, with its characteristic friction and drag, unlike the Euler equation, which models frictionless, inviscid flow. The Navier–Stokes equation has attracted much interest in various scientific systems, and examples of such systems are utilized to model and analyze the weather, ocean currents, water flow, pipe flow, and air flow. In addition, the Navier–Stokes equation, in its full and simplified form, is applied in regions ranging from the microscopic fluid study of blood flow [1,2] and the analysis of convective or diffusing pollution to the macroscopic design of power stations, the modelling of magneto-hydrodynamics, the design of aircraft and cars, and many other problems. Examples of models recently include the large-eddy simulations [3,4] and the fluid–particle coupling methods [5], and the cut-cell and ghost-cell methods [6,7,8].

On the other hand, the theoretical formulation of turbulence has involved plausibility and approximation for the compressible fluids [9] for over eight decades. Wyld [10] formulated the theory of turbulence, introducing a method of systematic perturbation similar to quantum field theory. Lee [11] described the generalization of Wyld’s formulation to the hydromagnetic equations in the theory of stationary, homogeneous, isotropic turbulence in compressible fluids and the solution for velocity and magnetic fields in the form of perturbation series. In the past, Forster, Nelson, and Stephen [12,13] have argued that the renormalization group theory applies to large-displacement, long-duration behavior of velocity correlations generalized by the Navier–Stokes equation for a randomly incompressible fluid. In particular, to obtain their results, they applied a forced Burger’s equation in one dimension, even though the physical understanding of the Navier–Stokes equation below two dimensions is not clear. As popular and well-known models, Antonov et al. [14,15] coupled the Kazantsev–Kraichnan velocity ensemble describing the environment to three different models: the Kardar–Parisi–Zhang (including Navier–Stokes equation) model, the Hwa–Kardar model, and the Pavlik model. They have shown a significant effect that leads to the induction of a non-linearity or the generation of an anisotropic scaling via the field theoretical renormalization group analysis. Meanwhile, Navier–Stokes turbulence [16] has been receiving attention in two-dimensional dynamical experiments for many years. Examples regarding two-dimensional Navier–Stokes turbulence are soap film flow [17,18], rotating fluids [19], magnetically forced stratified fluids [20], two-fluid hydrodynamics [21], and plasma in the equatorial ionosphere [22]. The fractional Brownian diffusion equation motion [23], the fractional generalized Langevin equation, and Fokker–Planck equations [24,25,26,27,28,29] have been used to theoretically and numerically discuss and analyze the motion of passive and active particles.

In this paper, we derive the Fokker–Planck equation. In Section 2, we find the joint probability density in the limits of t<<τ, t>>τ and for τ=0, where τ is correlation time [30]. In Section 3, the kurtosis, the correlation coefficient, the entropy, the combined entropy, and the moment-from-moment equation are numerically calculated. In Section 4, we provide an account of our conclusions, summarizing our key findings.

## 2. Navier–Stokes Equation with Harmonic Force and Magnetic Fields

### 2.1. Fokker–Planck Equation in Velocity and Magnetic Field

In this Subsection, we derive the Fokker–Planck equation for a conducting fluid in a magnetic field [10,11,16]. The modified equations of motion are expressed as follows:

(1) $$\frac{\partial h}{\partial t}=g_{h}(t),\frac{d}{dt}v(t)+v(t)\cdot \nabla v(t)-h(t)\cdot \nabla h(t)=e\nabla^{2}v(t)-r_{1}v(t)-\beta x(t)+g_{v}(t),$$

(2) $$\frac{\partial v}{\partial t}=-kx+g_{v}(t),\frac{d}{dt}h(t)+h(t)\cdot \nabla v(t)-v(t)\cdot \nabla h(t)=\varepsilon \nabla^{2}v(t)-r_{2}h(t)+g_{h}(t).$$

We introduce the incompressibility condition ∇⋅v(t)=0, and we apply this condition to Equations (1) and (2). Here, the parameters e and ε denote the drag coefficients, and $-r_{1}v(t)$ and $-r_{2}h(t)$ the viscous forces. −βx(t) and −kx(t) denote the harmonic forces acting on the particle in the velocity field and the magnetic field, respectively. We add the random forces $g_{v}(t)$ and $g_{h}(t)$ that activate the motion of the particle via the fluctuation of the particle, as follows:

(3) $$<g_{i}(t)>=0,<g_{i}(t)g_{i}(t^{\prime })>=g_{0i}^{2}g(t-t^{\prime })\;\mathrm{for}\;i=v,h.$$

Here, $g(t-t^{\prime })=\frac{1}{2\tau }\exp(-\frac{\left|t-t^{\prime }\right|}{\tau })$, $g_{0v}^{2}=2r_{1}k_{B}T_{v}$, and $g_{0h}^{2}=2r_{2}k_{B}T_{h}$. The parameters $g_{0v}$ and $g_{0h}$ denote the coupling strengths, $r_{1}$ and $r_{2}$ denote the frictional constants, $T_{v}$ and $T_{h}$ denote temperatures, $k_{B}$ denotes the Boltzmann constant, and τ denotes the correlation time. Equations (1) and (2) are two hydro-magnetic equations governed by the velocity field and the magnetic field. Later, we will find the approximate solutions to these equations [31]. Manipulating the integrals [32,33] from Equations (1) and (2), the equations of motion for P(x,v,h,t)≡P can be derived as follows:

(4) $$\frac{\partial }{\partial t}P=-v\frac{\partial }{\partial x}P+\alpha_{2}a(t)\frac{\partial^{2}}{\partial h^{2}}P+\frac{d}{dv}v\frac{d}{dx}vP+h^{2}\frac{d}{dv}\frac{d}{dx}P-e\frac{d}{dv}\frac{d^{2}}{dx^{2}}vP+r_{1}\frac{d}{dv}vP\;+\beta x\frac{\partial }{\partial v}P-\alpha_{1}b(t)\frac{\partial^{2}}{\partial x\partial v}P+\alpha_{1}a(t)\frac{\partial^{2}}{\partial v^{2}}P,\;\;\;\;\;\;\;\;\;\;$$

(5) $$\frac{\partial }{\partial t}P=-v\frac{\partial }{\partial x}P+kx\frac{\partial }{\partial v}P-a_{1}d(t)\frac{\partial^{2}}{\partial x\partial v}P+a_{1}c(t)\frac{\partial^{2}}{\partial v^{2}}P+\frac{d}{dh}h\frac{d}{dx}vP-\frac{d}{dh}v\frac{d}{dx}hP\;-\varepsilon \frac{d}{dh}\frac{d^{2}}{dx^{2}}hP+r_{2}\frac{d}{dh}hP+a_{2}c(t)\frac{\partial^{2}}{\partial h^{2}}P.$$

Here,

(6) $$\alpha_{1}=a_{1}=g_{0v}^{2}/2,\alpha_{2}=a_{2}=g_{0h}^{2}/2,$$

(7) a(t)=c(t)=1−exp(−t/τ), b(t)=d(t)=(t+τ)exp(−t/τ)−τ.

The above equation is called the Fokker–Planck equation [31] for the Navier–Stokes equation.

In order to find the joint probability density, we introduce the triple Fourier transform:

(8) $$P(\xi ,\eta ,\nu ,t)=\int_{-\infty }^{+\infty }dx\int_{-\infty }^{+\infty }dv\int_{-\infty }^{+\infty }dh\exp(-i\xi x-i\eta v-i\nu h)P(x,v,h,t).$$

By Fourier-transforming Equations (4) and (5), these equations can be derived as

(9) $$\frac{\partial }{\partial t}P(\xi ,\nu ,\eta ,t)=[-\beta \nu \frac{\partial }{\partial \xi }+[\xi -r_{1}\nu -e\xi^{2}\nu ]\frac{\partial }{\partial \nu }+\xi \nu \frac{\partial^{2}}{\partial \nu^{2}}-\xi \nu \frac{\partial^{2}}{\partial \eta^{2}}]P(\xi ,\nu ,\eta ,t)\;+[\alpha_{1}b(t)\xi \eta -\alpha_{1}a(t)\nu^{2}-\alpha_{2}a(t)\eta^{2}]P(\xi ,\nu ,\eta ,t),$$

(10) $$\frac{\partial }{\partial t}P(\xi ,\nu ,\eta ,t)=[-k\nu \frac{\partial }{\partial \xi }+\xi \frac{\partial }{\partial \nu }-[\varepsilon \xi^{2}+r_{2}]\eta \frac{\partial }{\partial \eta }]P(\xi ,\nu ,\eta ,t)\;+[a_{1}d(t)\xi \eta -a_{1}c(t)\nu^{2}-a_{2}c(t)\eta^{2}]P(\xi ,\nu ,\eta ,t).$$

### 2.2. P(x,t), P(v,t) and P(h,t) in the Short-Time Domain

#### 2.2.1. P(x,t), P(v,t), and P(h,t) in Navier–Stokes Equation for the Velocity

In this Subsection, we will find P(x,t), P(v,t), and P(h,t) in the Navier–Stokes equation for velocity in a short time domain t<<τ. In order to obtain the special solutions for ξ,ν,η through variable separation from Equation (9), three equations of velocity must be used, as follows:

(11) $$\frac{\partial }{\partial t}P(\xi ,t)=-\beta \nu \frac{\partial }{\partial \nu }P(\xi ,t)+\frac{1}{3}[\alpha_{1}b(t)\xi \nu -\alpha_{1}a(t)\nu^{2}-\alpha_{2}a(t)\eta^{2}]P(\xi ,t)+fP(\xi ,t),$$

(12) $$\frac{\partial }{\partial t}P(\nu ,t)=(\xi -r_{1}\nu -e\xi^{2}\nu +\xi \nu D_{\nu })\frac{\partial }{\partial \nu }P(\nu ,t)\;\;\;\;\;\;\;\;\;\;+\frac{1}{3}[\alpha_{1}b(t)\xi \nu -\alpha_{1}a(t)\nu^{2}-\alpha_{2}a(t)\eta^{2}]P(\nu ,t)-\frac{f}{2}P(\nu ,t),$$

(13) $$\frac{\partial }{\partial t}P(\eta ,t)=-\xi \nu D_{\eta }\frac{\partial }{\partial \eta }P(\eta ,t)+[\frac{1}{3}[\alpha_{1}b(t)\xi \nu -\alpha_{1}a(t)\nu^{2}-\alpha_{2}a(t)\eta^{2}]-\frac{f}{2}]P(\eta ,t).$$

Here, $D_{\nu }\equiv \partial /\partial \nu$, and f is the separation constant. In the steady state, by introducing $\frac{\partial }{\partial t}P(\xi ,t)=0$ and $P(\xi ,t)\to P^{st}(\xi ,t)$, we acquire Equation (11):

(14) $$P^{st}(\xi ,t)=\exp[\frac{1}{3\beta \nu }\left[\alpha_{1}b(t)\nu \frac{\xi^{2}}{2}-\alpha_{1}a(t)\nu^{2}\xi -\alpha_{2}a(t)\eta^{2}\xi ]P(\xi ,t)+\frac{f}{2\beta \nu }\xi P(\xi ,t)\right].$$

In order to find the solutions of the joint functions for ξ from P(ξ,t)≡ $Q(\xi ,t)P^{st}(\xi ,t)$, we must obtain the distribution functions via the calculation including terms up to the order of $t^{2}/\tau^{2}$; that is,

(15) $$P(\xi ,t)=Q(\xi ,t)P^{st}(\xi ,t),$$

(16) $$Q(\xi ,t)=R(\xi ,t)Q^{st}(\xi ,t)=R(\xi ,t)\exp[-\frac{1}{3{\left(\beta \nu \right)}^{2}}[\alpha_{1}b\prime (t)\nu \frac{\xi^{3}}{6}-a\prime (t)[\alpha_{1}\nu^{2}-\alpha_{2}\eta^{2}]\frac{\xi^{2}}{2}],$$

(17) $$R(\xi ,t)=S(\xi ,t)R^{st}(\xi ,t)=S(\xi ,t)\exp[\frac{1}{3{\left(\beta \nu \right)}^{3}}[\alpha_{1}b\prime \prime (t)\nu \frac{\xi^{4}}{24}-a\prime \prime (t)[\alpha_{1}\nu^{2}-\alpha_{2}\eta^{2}]\frac{\xi^{3}}{6}],$$

(18) $$S(\xi ,t)=T(\xi ,t)S^{st}(\xi ,t)=T(\xi ,t)\exp[-\frac{1}{3{\left(\beta \nu \right)}^{4}}[\alpha_{1}b\prime \prime \prime (t)\nu \frac{\xi^{5}}{120}]].$$

Here a′(t)≡d/dt, and $a\prime \prime (t)\equiv d^{2}/dt^{2}$. By discarding terms proportional to $1/\tau^{3}$ and taking the solutions to be arbitrary functions of the variable t−ξ/βν, the arbitrary function T(ξ,t) becomes T(ξ,t)=Θ[t−ξ/βν]. Consequently, we find that

(19) $$P(\xi ,t)=\Theta [t-\xi /\beta \nu ]S^{st}(\xi ,t)R^{st}(\xi ,t)Q^{st}(\xi ,t)P^{st}(\xi ,t).$$

Using the method from Equation (14) to Equation (19), we can obtain the Fourier transforms of the probability density for ν,η from Equations (12) and (13):

(20) $$P(\nu ,t)=\Theta [t+\nu /(\xi -r_{1}\nu -e\xi^{2}\nu +\xi \nu D_{\nu })]S^{st}(\nu ,t)R^{st}(\nu ,t)Q^{st}(\nu ,t)P^{st}(\nu ,t),$$

(21) $$P(\eta ,t)=\Theta [t-\eta /\xi \nu D_{\eta }]S^{st}(\eta ,t)R^{st}(\eta ,t)Q^{st}(\eta ,t)P^{st}(\eta ,t).$$

Therefore, based on Equations (19)–(21), we can calculate that

(22) $$\begin{array}{cc} P(\xi ,\nu ,\eta ,t) & =P(\xi ,t)P(\nu ,t)P(\eta ,t)\\ & =\exp[-\frac{\alpha_{1}t^{4}}{4\tau }[1-\frac{\tau }{t}]\xi^{2}-\frac{\alpha_{1}t^{3}}{6\tau }[1+\frac{2\beta }{t}]\nu^{2}-\frac{\alpha_{2}t^{3}}{2\tau }[1-\frac{\tau }{t}]\eta^{2}]. \end{array}$$

Using the inverse Fourier transform, we acquire

(23) $$P(x,t)={\left[\pi \frac{\alpha_{1}t^{4}}{\tau }[1-\frac{\tau }{t}]\right]}^{-1/2}\exp[-\frac{\tau x^{2}}{\alpha_{1}t^{4}}{\left[1-\frac{\tau }{t}\right]}^{-1}],$$

(24) $$P(v,t)={\left[2\pi \frac{\alpha_{1}t^{3}}{3\tau }[1+\frac{2\beta }{t}]\right]}^{-1/2}\exp[-\frac{3\tau v^{2}}{2\alpha_{1}t^{3}}{\left[1+\frac{2\beta }{t}\right]}^{-1}],$$

(25) $$P(h,t)={\left[2\pi \frac{\alpha_{2}t^{3}}{\tau }[1-\frac{\tau }{t}]\right]}^{-1/2}\exp[-\frac{\tau h^{2}}{2\alpha_{2}t^{3}}{\left[1-\frac{\tau }{t}\right]}^{-1}].$$

The mean squared displacements for P(x,t) are given by

(26) $$<x^{2}(t)>=\frac{\alpha_{1}t^{4}}{2\tau }[1-\frac{\tau }{t}],<v^{2}(t)>=\frac{\alpha_{1}t^{3}}{3\tau }[1+\frac{2\beta }{t}],<h^{2}(t)>=\frac{\alpha_{2}t^{3}}{\tau }[1-\frac{\tau }{t}].$$

#### 2.2.2. P(x,t), P(v,t), and P(h,t) in Navier–Stokes Equation for the Magnetic Field

For t<<τ from Equation (10), in order to obtain the special solutions for ξ,ν,η through variable separation, three equations for the magnetic field must be employed, as follows:

(27) $$\frac{\partial }{\partial t}P(\xi ,t)=-k\nu \frac{\partial }{\partial \xi }P(\xi ,t)+\frac{1}{3}[a_{1}d(t)\xi \nu -a_{1}c(t)\nu^{2}-a_{2}c(t)\eta^{2}]P(\xi ,t)+hP(\xi ,t),$$

(28) $$\frac{\partial }{\partial t}P(\nu ,t)=\xi \frac{\partial }{\partial \nu }P(\nu ,t)+\frac{1}{3}[a_{1}d(t)\xi \nu -a_{1}c(t)\nu^{2}-a_{2}c(t)\eta^{2}]P(\nu ,t)-\frac{h}{2}P(\nu ,t),$$

(29) $$\frac{\partial }{\partial t}P(\eta ,t)=-(\varepsilon \xi^{2}\eta +r_{2}\eta )\frac{\partial }{\partial \eta }P(\eta ,t)+\frac{1}{3}[a_{1}d(t)\xi \nu -a_{1}c(t)\nu^{2}-a_{2}c(t)\eta^{2}]P(\eta ,t)-\frac{h}{2}P(\eta ,t).$$

In the steady state, by introducing $\frac{\partial }{\partial t}P(\nu ,t)=0$ and $P(\nu ,t)\to P^{st}(\nu ,t)$, we acquire Equation (28):

(30) $$P^{st}(\nu ,t)=\exp[\frac{1}{3\xi }\left[-a_{1}d(t)\xi \frac{\nu^{2}}{2}+a_{1}c(t)\frac{\nu^{3}}{3}+a_{2}c(t)\eta^{2}\nu ]P(\nu ,t)-\frac{h}{2\xi }\nu P(\nu ,t)\right]$$

In order to find the solutions of joint functions for ν from P(ν,t)≡$Q(\nu ,t)P^{st}(\nu ,t)$, we must obtain the distribution functions via the calculation including terms up to the order of $t^{2}/\tau^{2}$; that is,

(31) $$P(\nu ,t)=Q(\nu ,t)P^{st}(\nu ,t),$$

(32) $$Q(\nu ,t)=R(\nu ,t)Q^{st}(\nu ,t)=R(\nu ,t)\exp[\frac{1}{3\xi^{2}}[-a_{1}d\prime (t)\xi \frac{\nu^{3}}{6}+a_{1}c\prime (t)\frac{\nu^{4}}{12}+a_{2}c\prime (t)\eta^{2}\frac{\nu^{2}}{2}]],$$

(33) $$R(\nu ,t)=S(\nu ,t)R^{st}(\nu ,t)=S(\nu ,t)\exp[\frac{1}{3\xi^{3}}[-a_{1}d\prime \prime (t)\xi \frac{\nu^{4}}{24}+a_{1}c\prime \prime (t)\frac{\nu^{5}}{60}+a_{2}c\prime \prime (t)\eta^{2}\frac{\nu^{3}}{6}]],$$

(34) $$S(\nu ,t)=T(\nu ,t)S^{st}(\nu ,t)=T(\nu ,t)\exp[-\frac{1}{3\xi^{3}}a_{1}d\prime \prime \prime (t)\frac{\nu^{4}}{24}].$$

By discarding terms proportional to $1/\tau^{3}$ and taking the solutions to be arbitrary functions of the variable t+ν/ξ, the arbitrary function T(ξ,t) becomes T(ν,t)=Θ[t+ν/ξ]. Consequently, we find that

(35) $$P(\nu ,t)=\Theta [t+\nu /\xi ]S^{st}(\nu ,t)R^{st}(\nu ,t)Q^{st}(\nu ,t)P^{st}(\nu ,t).$$

Through the method from Equation (30) to Equation (35), we can obtain the Fourier transforms of probability density for ξ,η from Equations (28) and (29):

(36) $$P(\xi ,t)=\Theta [t-\xi /\varepsilon \nu ]S^{st}(\xi ,t)R^{st}(\xi ,t)Q^{st}(\xi ,t)P^{st}(\xi ,t),$$

(37) $$P(\eta ,t)=\Theta [t-\ln\eta /(\lambda \xi^{2}+r_{2})]S^{st}(\eta ,t)R^{st}(\eta ,t)Q^{st}(\eta ,t)P^{st}(\eta ,t).$$

Therefore, based on Equations (35)–(37), we can calculate that

(38) $$\begin{array}{cc} P(\xi ,\nu ,\eta ,t) & =P(\xi ,t)P(\nu ,t)P(\eta ,t)\\ & =\exp[-\frac{a_{1}t^{4}}{24\tau }[1-\frac{4t}{15\tau }]\xi^{2}-\frac{a_{1}t}{6\tau }[1-\frac{t}{3\tau }]\nu^{2}-\frac{a_{2}t^{2}}{2\tau }[1-\frac{t}{a_{2}\tau }]\eta^{2}]. \end{array}$$

By using the inverse Fourier transform, we acquire

(39) $$P(x,t)={\left[\pi \frac{a_{1}t^{4}}{6\tau }[1-\frac{4t}{15\tau }]\right]}^{-1/2}\exp[-\frac{6\tau x^{2}}{a_{1}t^{4}}{\left[1-\frac{4t}{15\tau }\right]}^{-1}],$$

(40) $$P(v,t)={\left[2\pi \frac{a_{1}t}{3\tau }[1-\frac{t}{3\tau }]\right]}^{-1/2}\exp[-\frac{3\tau v^{2}}{2a_{1}t}{\left[1-\frac{t}{3\tau }\right]}^{-1}],$$

(41) $$P(h,t)={\left[2\pi \frac{a_{2}t^{2}}{\tau }[1-\frac{t}{a_{2}\tau }]\right]}^{-1/2}\exp[-\frac{\tau h^{2}}{2a_{2}t^{3}}{\left[1-\frac{t}{a_{2}\tau }\right]}^{-1}].$$

In the limit of t<<τ, the mean squared displacement, velocity, and magnetic field for P(v,t), P(v,t), and P(h,t), respectively, are given by

(42) $$<x^{2}(t)>=\frac{a_{1}t^{4}}{12\tau }[1-\frac{4t}{15\tau }],<v^{2}(t)>=\frac{a_{1}t}{3\tau }[1-\frac{t}{3\tau }],$$

(43) $$<h^{2}(t)>=\frac{a_{2}t^{2}}{\tau }[1-\frac{t}{a_{2}\tau }].$$

### 2.3. P(x,t), P(v,t), and P(h,t) in the Long Time Domain

#### 2.3.1. P(x,t), P(v,t), and P(h,t) in Navier–Stokes Equation for Velocity

In this Subsection, we will find P(x,t), P(v,t), and P(h,t) in long time domain t>>τ. For the long time domain, we write three equations for ξ,ν,η from Equations (11)–(13):

(44) $$\frac{\partial }{\partial t}P_{\xi }(\xi ,t)≅\frac{1}{3}[\alpha_{1}b(t)\xi \nu -\alpha_{1}a(t)\nu^{2}-\alpha_{2}a(t)\eta^{2}]P_{\xi }(\xi ,t),$$

(45) $$\frac{\partial }{\partial t}P_{\nu }(\nu ,t)≅\frac{1}{3}[\alpha_{1}b(t)\xi \nu -\alpha_{1}a(t)\nu^{2}-\alpha_{2}a(t)\eta^{2}]P_{\nu }(\nu ,t),$$

(46) $$\frac{\partial }{\partial t}P_{\eta }(\eta ,t)≅\frac{1}{3}[\alpha_{1}b(t)\xi \nu -\alpha_{1}a(t)\nu^{2}-\alpha_{2}a(t)\eta^{2}]P_{\eta }(\eta ,t).$$

In the steady state, as $\frac{\partial }{\partial t}P(\xi ,t)=0$,$\frac{\partial }{\partial t}P(\nu ,t)=0$, and $\frac{\partial }{\partial t}P(\eta ,t)=0$, we set the probability density P(ξ,t), P(ν,t), and P(η,t) as $P^{st}(\xi ,t)$, $P^{st}(\nu ,t)$ and $P^{st}(\eta ,t)$. Then, we can calculate $P_{\xi }^{st}(\xi ,t)$, $P_{\eta }^{st}(\nu ,t)$ and $P_{\eta }^{st}(\eta ,t)$ as follows:

(47) $$P_{\xi }^{st}(\xi ,t)=\exp[\frac{1}{3}\int \left[\alpha_{1}b(t)\xi \nu -\alpha_{1}a(t)\nu^{2}-\alpha_{2}a(t)\eta^{2}\right]dt],$$

(48) $$P_{\nu }^{st}(\nu ,t)=\exp[\frac{1}{3}\int \left[\alpha_{1}b(t)\xi \nu -\alpha_{1}a(t)\nu^{2}-\alpha_{2}a(t)\eta^{2}\right]dt],$$

(49) $$P_{\eta }^{st}(\eta ,t)=\exp[\frac{1}{3}\int \left[\alpha_{1}b(t)\xi \nu -\alpha_{1}a(t)\nu^{2}-\alpha_{2}a(t)\eta^{2}\right]dt].$$

Here, ∫a(t)dt=t−τ, a(t)=1, and ∫b(t)dt=−τt, b(t)=−τ in the long time domain. We continuously find $Q_{\xi }^{st}(\xi ,t)$,$Q_{\nu }^{st}(\nu ,t)$, and $Q_{\eta }^{st}(\eta ,t)$ for ξ,ν,η based on $P_{\xi }(\xi ,t)\equiv$ $Q_{\xi }(\xi ,t)P_{\xi }^{st}(\xi ,t)$:

(50) $$Q_{\xi }^{st}(\xi ,t)=\exp[\frac{1}{3}\int \left[-\alpha_{1}b(t)\xi \nu +\alpha_{1}a(t)\nu^{2}+\alpha_{2}a(t)\eta^{2}\right]dt],$$

(51) $$Q_{\nu }^{st}(\nu ,t)=\exp[\frac{1}{3}\int \left[-\alpha_{1}b(t)\xi \nu +\alpha_{1}a(t)\nu^{2}+\alpha_{2}a(t)\eta^{2}\right]dt],$$

(52) $$Q_{\eta }^{st}(\eta ,t)=\exp[\frac{1}{3}\int \left[-\alpha_{1}b(t)\xi \nu +\alpha_{1}a(t)\nu^{2}+\alpha_{2}a(t)\eta^{2}\right]dt].$$

Based on Equation (11) for ξ in a long time domain, the Fourier transform of the probability density in the steady state is calculated as $P^{st}(\xi ,t)=\exp[\frac{1}{3\beta \nu }[$$\alpha_{1}b(t)\nu \frac{\xi^{2}}{2}-a(t)[\alpha_{1}\nu^{2}-\alpha_{2}\eta^{2}]\xi ]$$+\frac{D_{\nu }}{3{\left(\beta \nu \right)}^{2}}$$\left[\alpha_{1}b(t)\eta \frac{\xi^{3}}{3}-a(t)[\alpha_{1}\nu^{2}-\alpha_{2}\eta^{2}]\frac{\xi^{2}}{2}\right]$. In the long time domain, it is clear that the Fourier transform of probability density $P^{st}(\xi ,t)$ for ξ is the same as Equation (14). Therefore, the probability density P(ξ,t) is given by

(53) $$P(\xi ,t)=\Theta [t-\xi /\beta \nu ]Q_{\xi }^{st}(\xi ,t)P^{st}(\xi ,t).$$

By substituting the calculated values of Equations (14) and (50) into Equation (53), we acquire P(ξ,t) of Equation (53). Using the similar method, we also acquire the Fourier transforms of probability density for ν,η:

(54) $$P(\nu ,t)=\Theta [t+\nu /(\xi -r_{1}\nu -e\xi^{2}\nu +\xi \nu D_{\nu })]Q_{\nu }^{st}(\nu ,t)P^{st}(\nu ,t),$$

(55) $$P(\eta ,t)=\Theta [t-\eta /\xi \nu D_{\eta }]Q_{\eta }^{st}(\eta ,t)P^{st}(\eta ,t).$$

For the long time domain t>>τ, from Equations (53)–(55), we can acquire

(56) $$\begin{array}{cc} P(\xi ,\nu ,\eta ,t) & =P(\xi ,t)P(\nu ,t)P(\eta ,t)\\ & =\exp[-\frac{2\alpha_{1}t}{3}[1+\tau ]\xi^{2}-\frac{2\alpha_{1}\beta^{2}t^{3}}{9}[1-\frac{3\tau }{\beta^{2}t}]\nu^{2}-\alpha_{2}t\eta^{2}]. \end{array}$$

Using the inverse Fourier transform, three probability densities can be calculated:

(57) $$P(x,t)={\left[2\pi \frac{4\alpha_{1}t}{3}[1+\tau ]\right]}^{-1/2}\exp[-\frac{3x^{2}}{8\alpha_{1}t}{\left[1+\tau \right]}^{-1}],$$

(58) $$P(v,t)={\left[2\pi \frac{4\alpha_{1}\beta^{2}t^{3}}{9}[1-\frac{3\tau }{\beta^{2}t}]\right]}^{-1/2}\exp[-\frac{9v^{2}}{8\alpha_{1}\beta^{2}t^{3}}{\left[1-\frac{3\tau }{\beta^{2}t}\right]}^{-1}],$$

(59) $$P(h,t)={\left[4\pi \alpha_{2}t\right]}^{-1/2}\exp[-\frac{h^{2}}{4\alpha_{2}t}].$$

The mean squared displacement, the mean squared velocity, and the mean squared magnetic field for P(x,t), P(v,t), and P(h,t), respectively, are given by

(60) $$<x^{2}(t)>=\frac{4\alpha_{1}t}{3}[1+\tau ],<v^{2}(t)>=\frac{4\alpha_{1}\beta^{2}t^{3}}{9}[1-\frac{3\tau }{\beta^{2}t}],<h^{2}(t)>=2\alpha_{2}t.$$

#### 2.3.2. P(x,t), P(v,t), and P(h,t) for the Magnetic Field

In the long time domain, we create approximately three equations for ξ,ν,η based on Equations (27)–(29):

(61) $$\frac{\partial }{\partial t}P_{\xi }(\xi ,t)≅\frac{1}{3}[a_{1}d(t)\xi \nu -a_{1}c(t)\nu^{2}-a_{2}c(t)\eta^{2}]P_{\xi }(\xi ,t),$$

(62) $$\frac{\partial }{\partial t}P_{\nu }(\nu ,t)≅\frac{1}{3}[a_{1}d(t)\xi \nu -a_{1}c(t)\nu^{2}-a_{2}c(t)\eta^{2}]P_{\nu }(\nu ,t),$$

(63) $$\frac{\partial }{\partial t}P_{\eta }(\eta ,t)≅\frac{1}{3}[a_{1}d(t)\xi \nu -a_{1}c(t)\nu^{2}-a_{2}c(t)\eta^{2}]P_{\eta }(\eta ,t).$$

In the steady state, by introducing $\frac{\partial }{\partial t}P_{\xi }(\xi ,t)=0$ for ξ from Equation (61) and $P_{\xi }(\xi ,t)\to P_{\xi }^{st}(\xi ,t)$, we can then calculate $P_{\xi }^{st}(\xi ,t)$, $P_{\eta }^{st}(\nu ,t)$ and $P^{st}(\eta ,t)$ as follows:

(64) $$P_{\xi }^{st}(\xi ,t)=\exp[\frac{1}{3}\int \left[a_{1}d(t)\xi \nu -a_{1}c(t)\nu^{2}-a_{2}c(t)\eta^{2}\right]dt],$$

(65) $$P_{\nu }^{st}(\nu ,t)=\exp[\frac{1}{3}\int \left[a_{1}d(t)\xi \nu -a_{1}c(t)\nu^{2}-a_{2}c(t)\eta^{2}\right]dt],$$

(66) $$P_{\eta }^{st}(\eta ,t)=\exp[\frac{1}{3}\int \left[a_{1}d(t)\xi \nu -a_{1}c(t)\nu^{2}-a_{2}c(t)\eta^{2}\right]dt].$$

We can find $Q_{\xi }^{st}(\xi ,t)$, $Q_{\nu }^{st}(\nu ,t)$, and $Q_{\eta }^{st}(\eta ,t)$ for ξ,ν,η using $P_{\xi }(\xi ,t)\equiv$ $Q_{\xi }(\xi ,t)P_{\xi }^{st}(\xi ,t)$:

(67) $$Q_{\xi }^{st}(\xi ,t)=\exp[\frac{1}{3}\int \left[-a_{1}d(t)\xi \nu +a_{1}c(t)\nu^{2}+a_{2}c(t)\eta^{2}\right]dt],$$

(68) $$Q_{\nu }^{st}(\nu ,t)=\exp[\frac{1}{3}\int \left[-a_{1}d(t)\xi \nu +a_{1}c(t)\nu^{2}+a_{2}c(t)\eta^{2}\right]dt],$$

(69) $$Q_{\eta }^{st}(\eta ,t)=\exp[\frac{1}{3}\int \left[-a_{1}b(t)\xi \nu +a_{1}a(t)\nu^{2}+a_{2}a(t)\eta^{2}\right]dt].$$

Based on Equation (28) for the magnetic field, the Fourier transform of the probability density for v is given by $P^{st}(\nu ,t)=\exp[\frac{1}{3\xi }$$[-a_{1}d(t)\xi \frac{\nu^{2}}{2}+a_{1}c(t)\frac{\nu^{3}}{3}$$+a_{2}c(t)\eta^{2}\nu ]P(\nu ,t)-\frac{h}{2\xi }\nu P(\nu ,t)]$ in the steady state. It is also clear in the long-time domain that the Fourier transform $P^{st}(\nu ,t)$ of the steady probability density for velocity is the same as Equation (30). Based on Equations (30) and (68), the probability density P(ν,t) is expressed as follows:

(70) $$P(\nu ,t)=\Theta [t+\nu /\xi ]Q_{\nu }^{st}(\nu ,t)P^{st}(\nu ,t).$$

Using a similar method, we can obtain the Fourier transforms of probability density for ξ, η as

(71) $$P(\xi ,t)=\Theta [t-\xi /\varepsilon \nu ]Q_{\xi }^{st}(\varsigma ,t)P^{st}(\xi ,t),$$

(72) $$P(\eta ,t)=\Theta [t-\ln\eta /(\lambda \xi^{2}+r_{2})]Q_{\eta }^{st}(\eta ,t)P^{st}(\eta ,t).$$

For the long-time domain t>>τ, from Equations (70)–(72), we have

(73) $$\begin{array}{cc} P(\xi ,\nu ,\eta ,t) & =P(\xi ,t)P(\nu ,t)P(\eta ,t)\\ & =\exp[-\frac{2a_{2}\tau t^{2}}{3}[1+\frac{a_{1}}{2a_{2}t}]\xi^{2}-\frac{2a_{1}\varepsilon^{2}t^{3}}{9}[1-\frac{3\tau }{\varepsilon^{2}t}]\nu^{2}-\frac{a_{2}t}{3}\eta^{2}]. \end{array}$$

Using the inverse Fourier transform, we can calculate three probability densities from Equation (73):

(74) $$P(x,t)={\left[2\pi \frac{4a_{2}\tau t^{2}}{3}[1+\frac{a_{1}}{2a_{2}t}]\right]}^{-1/2}\exp[-\frac{3x^{2}}{8a_{2}\tau t^{2}}{\left[1+\frac{a_{1}}{2a_{2}t}\right]}^{-1}],$$

(75) $$P(v,t)={\left[2\pi \frac{4a_{1}\varepsilon^{2}t^{3}}{9}[1-\frac{3\tau }{\varepsilon^{2}t}]\right]}^{-1/2}\exp[-\frac{9v^{2}}{8a_{1}\varepsilon^{2}t^{3}}{\left[1-\frac{3\tau }{\varepsilon^{2}t}\right]}^{-1}],$$

(76) $$P(h,t)={\left[2\pi \frac{2a_{2}t}{3}\right]}^{-1/2}\exp[-\frac{3h^{2}}{4a_{2}t}].$$

The mean squared values for P(v,t), P(v,t), and P(h,t), respectively, are given as follows:

(77) $$<x^{2}(t)>=\frac{4a_{2}\tau t^{2}}{3}[1+\frac{a_{1}}{2a_{2}t}],<v^{2}(t)>=\frac{4a_{1}\varepsilon^{2}t^{3}}{9}[1-\frac{3\tau }{\varepsilon^{2}t}],<h^{2}(t)>=\frac{2a_{2}t}{3}.$$

### 2.4. P(x,t), P(v,t), and P(h,t) in τ=0

#### 2.4.1. P(x,t), P(v,t), and P(h,t) for the Velocity

In this Subsection, we will find P(x,t), P(v,t), and P(h,t) in time domain τ=0 (t→∞). In the τ=0 domain (a(t)=1, b(t)=0), we write the approximate equations derived from Equations (11)–(13) for ξ,ν,η as follows:

(78) $$\frac{\partial }{\partial t}P(\xi ,t)=-\beta \nu \frac{\partial }{\partial \xi }P(\xi ,t)-\frac{1}{3}[\alpha_{1}\nu^{2}+\alpha_{2}\eta^{2}]P(\xi ,t),$$

(79) $$\frac{\partial }{\partial t}P(\nu ,t)=(\xi -r_{1}\nu -e\xi^{2}\nu +\xi \nu D_{\nu })\frac{\partial }{\partial \nu }P(\nu ,t)-\frac{1}{3}[\alpha_{1}\nu^{2}+\alpha_{2}\eta^{2}]P(\xi ,t),$$

(80) $$\frac{\partial }{\partial t}P(\eta ,t)=-\xi \eta D_{\nu }\frac{\partial }{\partial \eta }P(\eta ,t)-\frac{1}{3}[\alpha_{1}\nu^{2}+\alpha_{2}\eta^{2}]P(\xi ,t).$$

In the steady state, we calculate the steady probability densities $P^{st}(\xi ,t)$, $P^{st}(\nu ,t)$, and $P^{st}(\eta ,t)$ as follows:

(81) $$P^{st}(\xi ,t)=\exp[-\int \frac{1}{3\beta \nu }[\alpha_{1}\nu^{2}+\alpha_{2}\eta^{2}]d\xi ],$$

(82) $$P^{st}(\nu ,t)=\exp[-\int \frac{1}{3(\xi -r_{1}\nu -e\xi^{2}\nu +\xi \nu D_{\nu })}[\alpha_{1}\nu^{2}+\alpha_{2}\eta^{2}]d\nu ],$$

(83) $$P^{st}(\eta ,t)=\exp[-\int \frac{1}{3\xi \eta D_{\nu }}[\alpha_{1}\nu^{2}+\alpha_{2}\eta^{2}]d\eta ].$$

We find P(ξ,t), P(ν,t), and P(η,t) as follows:

(84) $$P(\xi ,t)=\Theta_{\xi }[t-\xi /\beta \nu ]P^{st}(\xi ,t),$$

(85) $$P(\nu ,t)=\Theta_{\xi }[t+\nu /(\xi -r_{1}\nu -e\xi^{2}\nu +\xi \nu D_{\nu })]P^{st}(\nu ,t),$$

(86) $$P(\eta ,t)=\Theta [t-\eta /\xi \eta D_{\nu })]P^{st}(\eta ,t).$$

Thus, we can calculate P(ξ,η,t) based on Equations (84)–(86):

(87) $$P(\xi ,\eta ,t)=P(\xi ,t)P(\eta ,t)=\exp[-\frac{\alpha_{1}r_{1}t^{4}}{8}[1+\frac{4}{3r_{1}t}]\xi^{2}-\frac{\alpha_{1}t}{2}[1+\frac{1}{4r_{1}t}]\nu^{2}-\frac{\alpha_{2}t^{2}}{6}\eta^{2}].$$

Using the inverse Fourier transform, P(ξ,t), P(v,t), and P(h,t), are, respectively, expressed as

(88) $$P(x,t)={\left[\pi \frac{\alpha_{1}r_{1}t^{4}}{2}[1+\frac{4}{3r_{1}t}]\right]}^{-1/2}\exp[-\frac{2x^{2}}{\alpha_{1}r_{1}t^{4}}{\left[1+\frac{4}{3r_{1}t}\right]}^{-1}],$$

(89) $$P(v,t)={\left[2\pi \alpha_{1}t[1+\frac{1}{4r_{1}t}]\right]}^{-1/2}\exp[-\frac{v^{2}}{2\alpha_{1}t}{\left[1+\frac{1}{4r_{1}t}\right]}^{-1}],$$

(90) $$P(h,t)={\left[2\pi \frac{\alpha_{2}t^{2}}{3}\right]}^{-1/2}\exp[-\frac{3h^{2}}{2\alpha_{2}t^{2}}].$$

Thus, the mean-squared deviations are given by

(91) $$<x^{2}(t)>=\frac{\alpha_{1}r_{1}t^{4}}{4}[1+\frac{4}{3r_{1}t}],<v^{2}(t)>=\alpha_{1}t[1+\frac{1}{4r_{1}t}],<h^{2}(t)>=\frac{\alpha_{2}t^{2}}{3}.$$

#### 2.4.2. P(x,t), P(v,t), and P(h,t) for the Magnetic Field

In the τ=0 domain (c(t)=1, d(t)=0), we can write the approximate equations from Equations (27)–(29) for ξ, ν, η:

(92) $$\frac{\partial }{\partial t}P(\xi ,t)=-k\nu \frac{\partial }{\partial \xi }P(\xi ,t)-\frac{1}{3}[a_{1}\nu^{2}+a_{2}\eta^{2}]P(\xi ,t),$$

(93) $$\frac{\partial }{\partial t}P(\nu ,t)=\xi \frac{\partial }{\partial \nu }P(\nu ,t)-\frac{1}{3}[a_{1}\nu^{2}+a_{2}\eta^{2}]P(\nu ,t),$$

(94) $$\frac{\partial }{\partial t}P(\eta ,t)=-(\varepsilon \xi^{2}\eta +r_{2}\eta )\frac{\partial }{\partial \eta }P(\eta ,t)-\frac{1}{3}[a_{1}\nu^{2}+a_{2}\eta^{2}]P(\eta ,t).$$

In the steady state, we calculate $P^{st}(\xi ,t)$, $P^{st}(\nu ,t)$, and $P^{st}(\eta ,t)$ as follows:

(95) $$P^{st}(\xi ,t)=\exp[-\int \frac{1}{3k\nu }[a_{1}\nu^{2}+a_{2}\eta^{2}]d\xi ],$$

(96) $$P^{st}(\nu ,t)=\exp[\int \frac{1}{3\xi }[a_{1}\nu^{2}+a_{2}\eta^{2}]d\nu ],$$

(97) $$P^{st}(\eta ,t)=\exp[-\int \frac{1}{3(\varepsilon \xi^{2}+r_{2})\eta }[a_{1}\nu^{2}+a_{2}\eta^{2}]d\eta ].$$

We find that the forms of P(ξ,t), P(ν,t), and P(η,t) can be derived as follows:

(98) $$P(\xi ,t)=\Theta [t-\xi /k\nu ]P^{st}(\xi ,t),P(\nu ,t)=\Theta [t+\nu /\xi ]P^{st}(\nu ,t),$$

(99) $$P(\eta ,t)=\Theta [t-\ln\eta /(\varepsilon \xi^{2}+r_{2})]P^{st}(\eta ,t).$$

Therefore, we can calculate P(ξ,η,t) from Equations (98) and (99) as follows:

(100) $$P(\xi ,\eta ,t)=P(\xi ,t)P(\eta ,t)=\exp[-\frac{a_{1}t^{3}}{18}\xi^{2}-\frac{a_{1}t}{2}\nu^{2}-\frac{a_{2}t}{3}\eta^{2}].$$

Using the inverse Fourier transform, P(x,t), P(v,t), and P(h,t), respectively, can be presented as

(101) $$P(x,t)={\left[2\pi \frac{a_{1}t^{3}}{9}\right]}^{-1/2}\exp[-\frac{9x^{2}}{2a_{1}t^{3}}],\;P(v,t)={\left[2\pi a_{1}t\right]}^{-1/2}\exp[-\frac{v^{2}}{2a_{1}t}],\;P(h,t)={\left[2\pi \frac{2a_{2}t}{3}\right]}^{-1/2}\exp[-\frac{3h^{2}}{4a_{2}t}].$$

From Equation (101), we can obtain the mean squared deviations:

(102) $$<x^{2}(t)>=\frac{a_{1}t^{3}}{9},<v^{2}(t)>=a_{1}t,<h^{2}(t)>=\frac{2a_{2}t}{3}.$$

## 3. Moment Equation, Kurtosis, Correlation Coefficient, Moment, and Entropy

We derive the moment equations [30] for $\mu_{l,m,n}^{v}$ and $\mu_{l,m,n}^{h}$ of velocity and the magnetic field as follows:

(103) $$\frac{d\mu_{l,m,n}^{v}}{dt}=(l-1)\mu_{l-1,m+1,n}+\alpha_{2}n(n-1)\mu_{l,m,n-2}+m^{2}\mu_{l,m,n}-2-l(l-1)\mu_{l-2,m+1,n+1}+eml(l-1)\mu_{l-2,m,n}\;-r_{1}m\mu_{l,m,n}-\beta \mu_{l+1,m-1,n}-\alpha_{1}b(t)lm\mu_{l-1,m-1,n}+\alpha_{1}a(t)m(n-1)\mu_{l,m-2,n},$$

(104) $$\frac{d\mu_{l,m,n}^{h}}{dt}=(l-1)\mu_{l-1,m+1,n}-km\mu_{l+1,m-1,n}-a_{1}d(t)lm\mu_{l-1,m-1,n}+a_{1}c(t)m(m-1)\mu_{l,m-2,n}+nl\mu_{l-1,m+1,n}\;+\varepsilon nl(l-1)\mu_{l-2,m,n-1}-r_{2}n\mu_{l,m,n}+a_{2}c(t)n(n-1)\mu_{l,m,n-2}.$$

Here, $\frac{d}{dt}\mu_{l,m,n}^{v}=\int_{-\infty }^{+\infty }dx\int_{-\infty }^{+\infty }dv\int_{-\infty }^{+\infty }dhP(x,v,h)x^{l}v^{m}h^{n}$. We calculate $\mu_{2,2,0},\;\mu_{2,0,2},\;\mu_{0,2,2}$ from Equations (103) and (104), and the other values will be published elsewhere.

The kurtosis for displacement and velocity, respectively, are given by

(105) $$K_{x}=<x^{4}>/3<x^{2}{>}^{2}-1,\;K_{v}=<v^{4}>/3<v^{2}{>}^{2}-1,\;K_{h}=<h^{4}>/3<h^{2}{>}^{2}-1.$$

We can write the correlation coefficients as

(106) $$\rho_{x,v}=<(x-<x>)(v-<v>)>/\sigma_{x}\sigma_{v},\;\rho_{x,h}=<(x-<x>)(h-<h>)>/\sigma_{v}\sigma_{h},\;\rho_{v,h}=<(v-<v>)(h-<h>)>/\sigma_{v}\sigma_{h}.$$

Here, we assume that a passive particle initially starts at $x=x_{0}$, $v=v_{0}$, and $h=h_{0}$ at time t=0. The parameters $\sigma_{v}$ and $\sigma_{h}$ are the root mean squared values for velocity and the magnetic field of joint probability density, respectively. Lastly, the entropies S(x,t) and S(v,t), respectively, can be calculated as follows:

(107) S(x,t)=−p(x,t)lnp(x,t), S(v,t)=−p(v,t)lnp(v,t).

The combined entropy is expressed as

(108) H(x,v,t)≡−∫dx∫dvp(x,t)p(v,t)lnp(x,t)p(v,t).

Entropy refers to a numerical representation of the reliability or quantity of information [34,35] that has a probability distribution; in a probability distribution, as the probability of a particular value increases and the probability of the remaining value decreases, the entropy decreases.

## 4. Summary

In summary, we have derived the Fokker–Planck equation in an incompressible conducting fluid induced by a magnetic field. We approximately obtained the solution of the joint probability density by using double Fourier transforms. In the hydro-magnetic case of two variables, i.e., velocity and a time-dependent magnetic field, the mean squared velocity for the joint probability density of velocity and a magnetic field has a super-diffusive form with time $∼t^{3}$ in t>>τ, while the mean squared displacement for the joint probability density of velocity and the magnetic field reduces to time $∼t^{4}$ in t<<τ. The motion of a passive particle for τ=0 and t>>τ behaves as a normal diffusion with a mean squared magnetic field $<h^{2}(t)>∼t$. In the short time domain t<<τ, the moment for an incompressible conducting fluid induced by a magnetic field becomes super-diffusive [36], with $\mu_{2,0,2}^{h}∼t^{6}$, consistent with our results. In particular, for τ=0, $\mu_{2,2,0}^{v}$ ($\mu_{2,0,2}^{v}$,$\mu_{0,2,2}^{v}$) scales to $∼t^{5}$ ($∼t^{6}$, $∼t^{3}$), consistent with our results. The combined entropy H(v,h,t) (H(h,v,t)) for an active particle on which the perturbation force acts has a minimum value of $∼\ln t^{2}$ ($∼\ln t^{2}$) in t>>τ (τ=0), while the largest displacement entropy value is proportional to $\ln t^{4}$ in t<<τ and τ=0. Table A1 of Appendix A summarizes the results of calculating the kurtosis, the correlation coefficient, the entropy, and the moment of probability density from the equations for two variables, the velocity field and the magnetic field, following the initial state $x=x_{0}$, $v=v_{0}$, $h=h_{0}$. Other values will be published elsewhere.

Despite great interest, the exact solutions for distributions of higher-order processes are rare, but an approximate solution of the probability distribution function is found in this paper. The approximate solution of the Navier–Stokes equations has not been solved to date, but our model provides a simple approximate solution through the Fourier transform. We hope to extend our model in the future to encompass the Navier–Stokes equation, adding the complexity of additional forces to our simulations for a more realistic representation of the system’s dynamics. Our findings allow for comparison and analysis against existing theories [37,38,39,40,41,42] computer simulations [43,44,45], and experimental data. The other results will be continuously published in other journals.

## Data Availability

Data will be made available on request.

## Appendix A

Table A1 summarizes the kurtosis, the correlation coefficient, and the moment in this paper.

**Table A1:** Approximate values of the kurtosis, the correlation coefficient, and the moment $\mu_{2,2,0},\mu_{2,0,2},\mu_{0,2,2}$ of the velocity field and the magnetic field in three time domains, where a passive particle initially starts at $x=x_{0}$, $v=v_{0}$, and $h=h_{0}$ at time t=0.

Time Domain	*v, h*	*x, v,* *h*	$K_{x},K_{v},K_{h}$	$\rho_{x,v},\rho_{x,h},\rho_{v,h}$	$\mu_{2,2,0},\mu_{2,0,2},\mu_{0,2,2}$	S(x,t),H(x,v,t) S(v,t),H(v,h,t) S(h,t),H(h,x,t)
t<<τ	*v*	*x*	$\frac{\tau^{2}x_{0}^{4}}{\alpha_{1}^{2}}t^{-8}+\frac{\tau x_{0}^{2}}{\alpha_{1}}t^{-4}$	$\rho_{x,v}=\frac{\tau x_{0}v_{0}}{\alpha_{1}}t^{-7/2}$	$\mu_{2,2,0}^{v}=\frac{\alpha_{1}^{2}}{\tau^{2}}t^{6}$ $\mu_{2,2,0}^{h}=\frac{\alpha_{1}^{3}}{\tau^{3}}t^{6}$	$\ln\frac{\alpha_{1}t^{4}}{\tau },\ln\frac{\alpha_{1}^{2}t^{7}}{\tau^{2}}$
		*v*	$\frac{\tau^{2}v_{0}^{4}}{\alpha_{1}^{2}}t^{-6}+\frac{\tau v_{0}^{2}}{\alpha_{1}}t^{-3}$	$\rho_{x,h}=\frac{\tau x_{0}h_{0}}{{\left(\alpha_{1}\alpha_{2}\right)}^{1/2}}t^{-3}$		$\ln\frac{\alpha_{1}t^{3}}{\tau },\ln\frac{\alpha_{1}\alpha_{2}t^{6}}{\tau^{2}}$
		*h*	$\frac{\tau^{2}h_{0}^{4}}{\alpha_{2}^{2}}t^{-6}+\frac{\tau h_{0}^{2}}{\alpha_{2}}t^{-3}$	$\rho_{v,h}=\frac{\tau x_{0}h_{0}}{{\left(\alpha_{1}\alpha_{2}\right)}^{1/2}}t^{-7/2}$	$\mu_{2,0,2}^{v}=\frac{\alpha_{1}\alpha_{2}}{\tau^{2}}t^{7}$ $\mu_{2,0,2}^{h}=\frac{\alpha_{1}^{2}\alpha_{2}}{\tau^{3}}t^{6}$	$\ln\frac{\alpha_{2}t^{3}}{\tau },\ln\frac{\alpha_{1}\alpha_{2}t^{7}}{\tau^{2}}$
	*h*	*x*	$\frac{\tau^{2}x_{0}^{4}}{\alpha_{1}^{2}}t^{-8}+\frac{\tau x_{0}^{2}}{\alpha_{1}}t^{-4}$	$\rho_{x,v}=\frac{\tau x_{0}v_{0}}{\alpha_{1}}t^{-5/2}$		$\ln\frac{\alpha_{1}t^{3}}{\tau },\ln\frac{\alpha_{1}^{2}t^{4}}{\tau^{2}}$
		*v*	$\frac{\tau^{2}v_{0}^{4}}{\alpha_{1}^{2}}t^{-2}+\frac{\tau v_{0}^{2}}{\alpha_{1}}t^{-1}$	$\rho_{x,h}=\frac{\tau x_{0}h_{0}}{{\left(\alpha_{1}\alpha_{2}\right)}^{1/2}}t^{-3}$	$\mu_{0,2,2}^{v}=\frac{\alpha_{1}\alpha_{2}}{\tau^{2}}t^{5}$ $\mu_{0,2,2}^{h}=\frac{\alpha_{1}^{2}a_{2}}{\tau^{3}}t^{5}$	$\ln\frac{\alpha_{1}t}{\tau },\ln\frac{\alpha_{1}\alpha_{2}t^{3}}{\tau^{2}}$
		*h*	$\frac{\tau^{2}h_{0}^{4}}{\alpha_{2}^{2}}t^{-4}+\frac{\tau h_{0}^{2}}{\alpha_{2}}t^{-2}$	$\rho_{v,h}=\frac{\tau v_{0}h_{0}}{{\left(\alpha_{1}\alpha_{2}\right)}^{1/2}\varepsilon }t^{-3/2}$		$\ln\frac{\alpha_{2}t^{2}}{\tau },\ln\frac{\alpha_{1}\alpha_{2}t^{5}}{\tau^{2}}$
t>>τ	*v*	*x*	$\frac{x_{0}^{4}}{\alpha_{1}^{2}}t^{-2}+\frac{x_{0}^{2}}{\alpha_{1}}t^{-1}$	$\rho_{x,v}=\frac{x_{0}v_{0}}{\alpha_{1}\beta }t^{-2}$	$\mu_{2,2,0}^{v}=\frac{e\alpha_{1}\beta^{2}}{\left(r_{1}-2\right)}t^{3}$ $\mu_{2,2,0}^{h}=\alpha_{1}^{2}t^{4}$	$\ln\alpha_{1}t,\ln\alpha_{1}^{2}\alpha_{2}\beta^{2}t^{2}$
		*v*	$\frac{v_{0}^{4}}{\alpha_{1}^{2}\beta^{4}}t^{-2}+\frac{v_{0}^{2}}{\alpha_{1}\beta^{2}}t^{-1}$	$\rho_{x,h}=\frac{\tau x_{0}h_{0}}{{\left(\alpha_{1}\alpha_{2}\right)}^{1/2}\beta }t^{-2}$		$\ln\alpha_{1}\beta^{2}t,\ln\frac{\alpha_{1}\alpha_{2}\beta^{2}t^{2}}{\tau }$
		*h*	$\frac{\tau^{2}h_{0}^{4}}{\alpha_{2}^{2}}t^{-2}+\frac{\tau h_{0}^{2}}{\alpha_{2}}t^{-1}$	$\rho_{v,h}=\frac{v_{0}h_{0}}{{\left(\alpha_{1}\alpha_{2}\right)}^{1/2}}t^{-2}$	$\mu_{2,0,2}^{v}=\alpha_{1}\alpha_{2}t^{2}$ $\mu_{2,0,2}^{h}=\frac{\alpha_{1}\alpha_{2}}{r_{2}}t^{3}$	$\ln\frac{\alpha_{2}t}{\tau },\ln\frac{\alpha_{1}\alpha_{2}t^{2}}{\tau }$
	*h*	*x*	$\frac{x_{0}^{4}}{\alpha_{1}^{2}\tau^{2}}t^{-8}+\frac{x_{0}^{2}}{\alpha_{1}\tau }t^{-4}$	$\rho_{x,v}=\frac{\tau x_{0}v_{0}}{{\left(\alpha_{1}\alpha_{2}\tau \right)}^{1/2}\varepsilon }t^{-5/2}$		$\ln\alpha_{1}\tau t^{2},\ln\alpha_{1}^{2}\varepsilon^{2}\tau t^{5}$
		*v*	$\frac{v_{0}^{4}}{\alpha_{1}^{2}\varepsilon^{4}}t^{-6}+\frac{v_{0}^{2}}{\alpha_{1}\varepsilon^{2}}t^{-3}$	$\rho_{x,h}=\frac{x_{0}h_{0}}{{\left(\alpha_{1}\alpha_{2}\tau \right)}^{1/2}}t^{-2}$	$\mu_{0,2,2}^{v}=\frac{\alpha_{1}\alpha_{2}\beta^{2}}{(r_{1}-2)\tau }t^{3}$ $\mu_{0,2,2}^{h}=\frac{\alpha_{1}\alpha_{2}}{r_{2}\tau }t^{2}$	$\ln\alpha_{1}\varepsilon^{2}t^{3},\ln\alpha_{1}\alpha_{2}\varepsilon^{2}t^{5}$
		*h*	$\frac{h_{0}^{4}}{\alpha_{2}^{2}}t^{-4}+\frac{h_{0}^{2}}{\alpha_{2}}t^{-2}$	$\rho_{v,h}=\frac{v_{0}h_{0}}{{\left(\alpha_{1}\alpha_{2}\right)}^{1/2}\varepsilon }t^{-5/2}$		$\ln\alpha_{2}t^{2},\ln\alpha_{1}\alpha_{2}\tau t^{4}$
τ=0	*v*	*x*	$\frac{x_{0}^{4}}{\alpha_{1}^{2}r_{1}^{2}}t^{-8}+\frac{x_{0}^{2}}{\alpha_{1}r_{1}}t^{-4}$	$\rho_{x,v}=\frac{x_{0}v_{0}}{\alpha_{1}r_{1}^{1/2}}t^{-5/2}$	$\mu_{2,2,0}^{v}=\frac{\alpha_{1}^{2}r_{1}}{\left(r_{1}-2\right)}t^{5}$ $\mu_{2,2,0}^{h}=\frac{\alpha_{1}^{3}}{\tau }t^{6}$	$\ln\alpha_{1}r_{1}t^{4},\ln\alpha_{1}^{2}r_{1}t^{5}$
		*v*	$\frac{v_{0}^{4}}{\alpha_{1}^{2}}t^{-2}+\frac{v_{0}^{2}}{\alpha_{1}}t^{-1}$	$\rho_{x,h}=\frac{\tau x_{0}h_{0}}{{\left(\alpha_{1}\alpha_{2}\right)}^{1/2}}t^{-3/2}$		$\ln\alpha_{1}t,\ln\alpha_{1}\alpha_{2}t^{3}$
		*h*	$\frac{h_{0}^{4}}{\alpha_{2}^{2}}t^{-4}+\frac{h_{0}^{2}}{\alpha_{2}}t^{-2}$	$\rho_{v,h}=\frac{v_{0}h_{0}}{{\left(\alpha_{1}\alpha_{2}\right)}^{1/2}r_{2}^{1/2}}t^{-3/2}$	$\mu_{2,0,2}^{v}=\frac{\alpha_{1}\alpha_{2}r_{1}}{\tau }t^{6}$ $\mu_{2,0,2}^{h}=\frac{\alpha_{1}\alpha_{2}^{2}}{r_{2}}t^{3}$	$\ln\alpha_{2}t^{2},\ln\alpha_{1}\alpha_{2}r_{1}t^{6}$
	*h*	*x*	$\frac{x_{0}^{4}}{\alpha_{1}^{2}}t^{-6}+\frac{x_{0}^{2}}{\alpha_{1}}t^{-3}$	$\rho_{x,v}=\frac{x_{0}v_{0}}{\alpha_{1}}t^{-2}$		$\ln\alpha_{1}t^{3},\ln\alpha_{1}^{2}t^{4}$
		*v*	$\frac{v_{0}^{4}}{\alpha_{1}^{2}}t^{-2}+\frac{v_{0}^{2}}{\alpha_{1}}t^{-1}$	$\rho_{x,h}=\frac{x_{0}h_{0}}{\alpha_{1}}t^{-2}$	$\mu_{0,2,2}^{v}=\frac{\alpha_{1}\alpha_{2}}{\left(r_{1}-2\right)}t^{3}$ $\mu_{0,2,2}^{h}=\frac{\alpha_{1}^{2}\alpha_{2}}{r_{2}\tau }t^{3}$	$\ln\alpha_{1}t,\ln\alpha_{1}\alpha_{2}t^{2}$
		*h*	$\frac{h_{0}^{4}}{\alpha_{2}^{2}}t^{-2}+\frac{h_{0}^{2}}{\alpha_{2}}t^{-1}$	$\rho_{v,h}=\frac{v_{0}h_{0}}{{\left(\alpha_{1}\alpha_{2}\right)}^{1/2}}t^{-2}$		$\ln\alpha_{2}t,\ln\alpha_{1}\alpha_{2}t^{4}$
